# Ovarian function suppression decision-making and uptake in premenopausal women with breast cancer: a mixed methods analysis

**DOI:** 10.1007/s10549-026-07929-1

**Published:** 2026-02-23

**Authors:** N. L. Henry, L. K. Monkman, K. Griffith, K. Scheu, T. Ghormley, J. Armstrong, M. Secor, D. Jasthi, S. T. Hawley, T. Guetterman

**Affiliations:** 1 Department of Internal Medicine, University of Michigan Medical School, Ann Arbor, MI USA; 2 Department of Family Medicine, University of Michigan Medical School, Ann Arbor, MI USA; 3 Department of Biostatistics, School of Public Health, University of Michigan, Ann Arbor, MI USA; 4 Michigan Medicine, Ann Arbor, MI USA

**Keywords:** Breast cancer, Premenopausal, Ovarian function suppression, Gonadotropin-releasing hormone agonist, Decision-making

## Abstract

**Purpose:**

Ovarian function suppression (OFS) reduces the risk of recurrence of hormone receptor-positive breast cancer but increases the likelihood of toxicity and nonpersistence with endocrine therapy. In addition, rates of OFS utilization are lower than expected. To increase understanding of these issues, we sought to identify patient factors associated with the use of OFS injections, as well as treatment decision-making and education needs.

**Methods:**

In this convergent mixed methods designed study, patients receiving OFS, who started then discontinued OFS injections, and who never initiated OFS injections underwent 1:1 semi-structured interviews and completed questionnaires on shared decision-making and medication beliefs.

**Results:**

Of 33 enrolled participants, 30 completed both the questionnaires and the interview. Median age was 43 (range 32–55), 24 were white (80%), and 20 (66.7%) had received chemotherapy. Four key themes emerged. (1) There was concern about need for more education, especially about short- and long-term side effects of OFS. (2) For those receiving OFS injections, the decision to take OFS was mainly due to a desire to reduce cancer recurrence risk. (3) For those who stopped OFS, injections were often used as a stop-gap measure, with a preference for permanence of oophorectomy. (4) For those who never took OFS, there was often perceived lack of strong physician recommendation.

**Conclusion:**

Tailored support for patients is needed to optimize decision-making regarding OFS, related to both potential benefits and risks of OFS in addition to adjuvant endocrine therapy. Educational strategies such as peer mentors or decision aids should be explored in this clinical setting.

## Introduction

Although the average age of breast cancer diagnosis in the United States (US) is approximately 62 years, more younger, pre- and perimenopausal women are being diagnosed [1–3]. In 2024, an estimated 50,830 women under age 50 were diagnosed with breast cancer in the US [4]. Between 2012 and 2021, rates of breast cancer in women under age 50 increased 1.4% per year, compared to 0.7% per year in older women [4]. The international SOFT and TEXT trials demonstrated that for premenopausal women with hormone receptor-positive (HR +) breast cancer, the addition of ovarian function suppression (OFS) to adjuvant endocrine therapy (ET) significantly improved disease-free survival compared to tamoxifen alone [5, 6]. In addition, for patients at higher risk of recurrence, the addition of OFS has been shown to numerically improve overall survival [5].

Since 2015, international guidelines have recommended the addition of OFS to adjuvant ET for pre- and perimenopausal patients at increased risk of recurrence of HR + breast cancer [7, 8]. Increased risk has been variably defined as age less than 35, treatment with chemotherapy, higher stage disease, and grade 3 tumors [7, 8]. However, the SOFT and TEXT trials also demonstrated that addition of OFS to ET increased patient-reported symptoms (e.g., vasomotor, gynecologic) and decreased quality of life, especially for those younger than age 35 [9, 10].

OFS is usually accomplished through administration of gonadotropin-releasing hormone agonist (GnRHa) injections such as goserelin or leuprolide, which are typically given in the clinic or infusion room every 4 or 12 weeks. Additional forms of OFS include bilateral salpingo-oophorectomy (BSO) or pelvic radiation, which can be used to ablate ovarian function.

Analysis of administrative pharmacy data from the US suggests low uptake of GnRHa therapy, with only about 50% of patients under the age of 45 who received chemotherapy for HR + breast cancer receiving OFS injections in 2021, with an additional 5% undergoing BSO [11]. A study of similar patients with moderate to high risk of breast cancer recurrence in Germany demonstrated that in 2023, 42% were recommended to receive OFS via any method plus aromatase inhibitor therapy [12]. Reasons for nonreceipt of OFS for eligible patients are uncertain and likely due to both clinician and patient factors. Through this convergent mixed methods designed study, we sought to identify patient factors associated with both OFS injection initiation and persistence, as well as specific treatment decision-making and education needs of premenopausal patients with HR + breast cancer.

## Methods

Between February and August 2025, participants were recruited from breast cancer clinics at a single academic medical center in Ann Arbor, MI (*n* = 24), and via the UMHealthResearch.org website (*n* = 9). Eligibility criteria included: (1) diagnosis of stage 1–3 HR + breast cancer within 3 years prior to study entry, (2) pre- or perimenopausal at the time of breast cancer diagnosis, (3) potential candidate for the addition of OFS injections to adjuvant ET according to the treating physician, (4) able to speak English, and (5) willing to provide informed consent and participate in an interview via video or telephone. The study was granted an exemption (exemption 2) from the University of Michigan IRBMED (HUM00262102). Participants provided verbal consent to participate after reviewing a written informed consent document. All participants who completed the questionnaires and interview described below were compensated with a $50 gift card.

Participants were included in one of three cohorts: (a) initiated treatment with OFS injections and still on treatment at the time of the interview, (b) initiated treatment with OFS but discontinued treatment for any reason by the time of the interview, and (c) never initiated treatment with OFS. Clinicians from the breast cancer program assisted with identification of patients; in addition, the electronic medical record was queried for prescription of goserelin or leuprolide. Recruitment for cohorts a and b stopped when thematic saturation was reached based on coder agreement that new codes were not being added; recruitment for cohort c stopped because of inability to identify additional participants.

Prior to participating in the 1:1 interview, participants were sent a link to complete four electronic questionnaires. One questionnaire collected self-reported demographic information (e.g., sex, race/ethnicity, marital status, education, annual income, height, weight, and cancer stage and treatment). The second was the 9-item Shared Decision-Making Questionnaire (SDM-Q-9), which asked about physicians’ and patients’ behavior related to the decision to take OFS injections, using a 6-point Likert scale [13]. The third was the Control Preferences Scale (CPS), a two-item questionnaire about (a) actual role and (b) preferred role in dealing with one’s cancer diagnosis. Each question had 5 levels of possible answers ranging from active (patient alone) to passive (physician alone) [14]. For the CPS, each question had 5 levels of possible answers ranging from active (patient alone) to passive (physician alone), and responses were categorized into patient-based, collaborative, and physician-based. The fourth was the 10-item Beliefs about Medication Questionnaire (BMQ), which queries personal views about the necessity of taking adjuvant ET to control their breast cancer and concerns about adverse effects from ET [15]. The BMQ’s two 5-item subscales each have a 5-item Likert answer option, and higher values reflect more of the concept being assessed. Questionnaires were scored according to published instructions [13, 15]. Questionnaire data were analyzed descriptively.

Semi-structured interviews lasting 30–60 min were conducted via video conferencing or telephone individually with participants. The interview guides for each cohort (Online Resource 1), which included questions and exploratory probes, were developed by DJ, NLH, TG, and LM; interviews were conducted by LM. Each interview was recorded and professionally transcribed verbatim. Two study team members (LM and TG) analyzed the interview data thematically, including developing the codebook and coding each transcript (MAXQDA). Discrepancies were resolved by consensus. Coded data were then synthesized into themes by examining patterns across codes, and representative quotes were identified.

We then conducted a mixed methods analysis to develop meta-interferences, findings that arise from integrating the results from the questionnaires with the themes from the qualitative study. Starting with the existing codebook, we imported the quantitative ratings into MAXQDA to create a new merged dataset, which we then analyzed using code reports from MAXQDA’s segment matrix tool. Comparing and contrasting questionnaire scores with excerpts from interviews with patients in the different cohorts allowed us to examine whether quantitative findings were consistent with the qualitative themes.

## Results

### Participant characteristics

Of the 33 patients who provided informed consent, 30 completed both the questionnaires and the interviews (Table 1). Median age was 43 years (range 32–55); 3 were Asian (10%), 3 Black (10%, one of whom was Hispanic), and 24 white (80%). About three-quarters of participants were married or in a committed relationship, and a similar number had at least a college education. Two-thirds had received chemotherapy. All had received treatment with ET since diagnosis, with 11 taking only tamoxifen, 16 taking only aromatase inhibitor therapy, and 3 taking both classes of ET. Two participants were treated at institutions other than University of Michigan. Two did not complete the interview, and one patient’s interview was excluded from analysis because she reported no discussion about OFS initiation.

**Table 1:** Characteristics of enrolled participants who completed electronic questionnaires

Characteristic	All Patients (*N* = 30)	On OFS (A), *n* = 13	Previously on OFS (B), *n* = 12	Never on OFS (C), *n* = 5
Age, median (range)	43 (32–55)	46 (32–55)	42 (34–51)	43 (35–44)
% ≤ 40 years old	10 (33.3%)	3 (23.1%)	5 (41.7%)	2 (40.0%)
Race/ethnicity				
Asian/NH	3 (10.0%)	2 (15.4%)	1 (8%)	0
Black/NH	2 (6.7%)	0	0	2 (40.0%)
Black/H	1 (3.3%)	1 (7.7%)	0	0
White/NH	24 (80.0%)	10 (76.9)	11 (92%)	3 (60.0%)
Partnered status				
Single	3 (10.0%)	3 (23.1%)	0	0
Married or committed	24 (80.0%)	8 (61.5%)	11 (91.7%)	5 (100.0%)
Divorced	3 (10.0%)	2 (15.4%)	1 (8.3%)	0
Education				
High school or less	3 (10.0%)	0	3 (25%)	0
Some college	4 (13.3%)	2 (15.4%)	2 (16.7%)	0
College degree	14 (46.7%)	5 (38.5%)	5 (41.7%)	4 (80.0%)
Postgraduate	9 (30.0%)	6 (46.2%)	2 (16.7%)	1 (20.0%)
Income				
< $30,000	5 (16.7%)	2 (15.4%)	3 (25%)	0
$30,001-$60,000	2 (6.7%)	0	2 (16.7%)	0
$60,001-$100,000	8 (26.7%)	3 (23.1%)	3 (25%)	2 (40.0%)
$100,000	13 (43.3%)	6 (46.2%)	4 (33.3%)	3 (60.0%)
Prefer not to say	2 (6.7%)	2 (15.4%)	0	0
Cancer stage				
Stage I	10 (33.3%)	3 (23.1%)	5 (41.7%)	2 (40.0%)
Stage II	13 (43.3%)	6 (46.2%)	4 (33.3%)	3 (60.0%)
Stage III	5 (16.7%)	3 (23.1%)	2 (16.7%)	0
Unknown	2 (6.7%)	1 (7.7%)	1 (8.3%)	0
Prior chemotherapy	20 (67.7%)	12 (92.3%)	5 (41.7%)	3 (60.0%)
Body mass index, mean (SD), kg/m^2^	27.6 (7.2)	28.1 (8.9)	28.1 (6.3)	25.4 (4.3)

*H* Hispanic, *NH* Non-Hispanic, *OFS* ovarian function suppression, *SD* standard deviation

Based on the questionnaire data, more than 90% of participants preferred either taking an active role in their decision-making or making the decision collaboratively with their oncologist (each 46.7%; Table 2). Four participants reported misalignment between actual and preferred decision-making preferences; two preferred active versus their actual collaborative process, and two preferred collaborative versus their actual active role. Of those still taking OFS, 23.1% reported taking an active role in the decision to take OFS; in contrast, 66.7% of those who stopped OFS injections and 60% who never initiated the therapy reported taking an active role. On the BMQ questionnaire, participant-reported necessity of the OFS medication was fairly well balanced with the concerns across the population, with numerically slightly higher concerns reported by the cohort that stopped OFS compared to those still taking it and those who never started taking OFS.

**Table 2:** Patient-reported questionnaire data

Characteristic	All patients (n = 30)	On OFS (A), *n* = 13	Previously on OFS (B), *n* = 12	Never on OFS (C), *n* = 5
Shared decision-making (SDM) Questionnaire-9 (0–100)	71.6 (26.2)	69.1 (29.7)	70 (22.9)	82.2 (26.7)
Actual role in SDM				
Active	14 (46.7%)	3 (23.1%)	8 (66.7%)	3 (60.0%)
Collaborative	14 (46.7%)	8 (61.5%)	4 (33.3%)	2 (40.0%)
Passive	2 (6.7%)	2 (15.4%)	0	0
Preferred role in SDM				
Active	14 (46.7%)	4 (30.8%)	7 (58.3%)	3 (60.0%)
Collaborative	14 (46.7%)	7 (53.8%)	5 (41.7%)	2 (40.0%)
Passive	2 (6.7%)	2 (15.4%)	0	0
Beliefs about medications questionnaire (BMQ)				
Necessity	11.6 (4.6)	12.2 (4.7)	10.9 (4.8)	11.8 (4.7)
Concern	11.2 (4.3)	10.7 (4.9)	12 (3.7)	10.6 (4.6)
Difference	0.4 (7.0)	1.5 (6.8)	− 1.1 (7.9)	1.2 (5.8)
BMQ				
Necessity outweighs Concern	13 (43.3%)	7 (53.9%)	3 (25%)	3 (60%)
Necessity equal to Concern	2 (6.7%)	1 (7.7%)	1 (8.3%)	0
Concern outweighs Necessity	15 (50%)	5 (38.5%)	8 (66.7%)	2 (40%)

*OFS* ovarian function suppression

### Qualitative themes regarding OFS decision-making across cohorts

Four different major themes were generated for participants in each of the three cohorts. (1) For those continuing to take OFS, the decision to take OFS was primarily due to a desire to reduce risk of cancer recurrence. (2) For those who stopped taking OFS injections, the injections were often seen as and employed as a stop-gap measure. Those patients expressed a desire for the permanence of oophorectomy, which they contrasted to the repeated need for injections during a long prescription time period. (3) For those who never started OFS injections, the decision not to take OFS was often due to a perceived lack of strong physician recommendation. Finally, (4) for all participants, concern about short- and long-term side effects of OFS, menopause more generally, and negative effects on quality of life was a consistent theme. Notably, all participants who decided against taking OFS injections reported taking tamoxifen; none reported declining all adjuvant ET. Table 3 includes examples of quotes for each of these themes.

**Table 3:** Qualitative Themes Regarding Ovarian Function Suppression (OFS) Decision-Making Across Cohorts

Theme	Representative quotes
OFS to reduce risk of cancer recurrence	“They told me that it gave me a lot of protection against getting cancer again in the future…since I’m more high risk and I’m younger, so I have a lot more life hopefully to live. I definitely wanted to do it.” (1625, still taking OFS) “**I guess if they said all your hair’s going to fall out and you’re going to be green or something, but I mean I would rather be bald and green than have cancer**” (8885, still taking OFS) “Whatever I need to do to get rid of it and prevent it from coming back, sign me up. It just was a no brainer to me.” (8843, still taking OFS)
Use of OFS injections until undergoing oophorectomy	“Ultimately I always knew that I was getting my ovaries removed, so the [OFS] was always going away. For one, I [didn’t] want to put that chemical in my body if I didn’t have to.” (8370, stopped OFS) **“I really didn’t think that I was going to make it multiple years on the shots, but I really didn’t want to go through a surgery a month after going through chemo and then a month before having another surgery.” (2949, stopped OFS)**
Concern about side effects	“I think I could have lived with the inconvenience for 10 years, but I don’t think I could have lived with the side effects for 10 years” (2949, stopped OFS) **“I’m learning more and more about all the things estrogen affects when I wake up every morning and my body feels nothing like what my body felt a year ago … that I’m feeling like I am 70 instead of 45.” (3549, stopped OFS)** “Now that I’ve been on it for a couple years now and have been part of some other discussions around being in early medically induced menopause, there are some concerns long-term about heart … brain fog … bone density… long-term at a young age” (0486, still taking OFS) **“[my oncologist] lets me know that it would be completely stopping my estrogen, and that for me was a big concern.” (2839, didn’t start OFS)**
Non-initiation of OFS because of lack of strong physician recommendation	**“side effects of doing the ovarian suppression would outweigh the benefits” (3020, didn’t start OFS)** “It wasn’t presented as a recommendation” (3337, didn’t start OFS)

Participant identification numbers are given in parentheses

*OFS to reduce risk of cancer recurrence.* Participants who received OFS injections often described the main reason they opted to take the treatment was to reduce their risk of breast cancer recurrence, on the recommendation of their physician. For example, one patient shared that reducing risk was “my main motivating factor. I don’t want breast cancer.” Many expressed that the potential benefit from the treatment would outweigh any concerns about side effects of the therapy, describing “if there are side effects, I wouldn’t have cared quite honestly.”

*Use of OFS injections until undergoing oophorectomy*. Numerous participants reflected on using OFS injections only as “a stop gap” until they underwent bilateral salpingo-oophorectomy (BSO). Several mentioned that the idea of ovary removal as “more permanent” was appealing, especially as many described the experience of receiving injections as an ordeal, due to the inconvenience and physical discomfort. One participant also noted that ovary removal would reduce the “chance of ovarian cancer.”

*Concern about side effects.* Most participants, especially those still taking OFS and a few who discontinued treatment, described their OFS side effects as “manageable.” However, a number of participants who had initiated then stopped OFS did so in part because they perceived toxicity as diminishing their quality of life. For example, one participant noted cardiac symptoms that she concluded were “because [she was] being pushed into menopause.” Others reported feeling much older than their chronological age after starting OFS injections. However, even for those who continued with OFS, most reported concern about long-term effects, including “brain fog,” “bone density,” and “heart health.” In addition, many participants remarked that these potential effects “weren’t necessarily discussed when I went on it.”

Similarly, participants who never initiated OFS reported concern about abruptly lowering estrogen levels, medication side effects, and the potential for long-term effects of OFS. For example, one participant questioned, “Are there long-term issues that you could get from it…?” One participant even noted, “we were pleasantly surprised that tamoxifen would not have the same necessarily dramatic effect on bone loss…”.

*Non-initiation of OFS because of lack of strong physician recommendation*. Two of the four participants who did not initiate OFS noted that the discussion with the oncologist about OFS treatment was very brief. One mentioned “it seemed like…it was an option, but not necessary.” Another stated her oncologist said “she wouldn’t necessarily recommend it for my case.”

### Patient-centered education and decision-making in the context of receiving OFS

The qualitative interviews highlighted that participants closely associated decision-making about taking OFS as part of cancer treatment with patient education needs. One theme was patient-centered decision-making support, and relatedly, patient information preferences. Finally, a theme emerged related to the specific information needs of those diagnosed at age 40 or younger. Table 4 includes examples of quotes for each theme.

**Table 4:** Patient-Centered Education and Decision-Making in the Context of Ovarian Function Suppression

Theme	Representative Quotes
Decision-making support	“…I would have liked somebody to say to me, listen, you have some medical decisions coming up and I don’t know how you like to make medical decisions but I could give you a lot of detailed information” (5487) **“Have you guys considered having patient advocates…to ask questions? Why did you make the decision you did? …just to share the experience to help them as they kind of walk through…someone that has the experience” (3771)**
Information preferences	“There’s so much information just kind of piled on you with different side effects from chemo, radiation, and then being on the ovarian suppression, so it’s just overwhelming” (1034) **“…once they’ve had a little bit of time to process the information, experience things…that information can be better incorporated, that there can be more opportunities for those types of discussions” (0240)** ***Information about the OFS injection itself:*** “It’s a rather large needle…this isn’t a normal shot, the needle is larger, it’s going to actually put something in your body. I didn’t even know that.” (8370) **“Potentially one piece of information … that I think could be helpful, especially in terms of the pain…is options for mitigating the pain or discomfort with the shots” (7758)**
Information needs of patients under age 40	“under 40 your body’s not supposed to be experiencing those things … is it a feeling of your life just got cut short … it does change some relationships and the way you interact with people” (0486) **“[for women] with breast cancer that are under the age of 40… there’s just not a lot of information about what this means and the long-term impacts of not having estrogen in your body. It is scary.” (2949)** “I am definitely in some young adult groups … having a group chat or a community where if I’m having an issue and I ask about it and then find out if … other people are having the same, that also helps” (3084)

Participant identification numbers are given in parentheses

*Decision-making support*: Some participants recommended first asking patients about their medical information and decision-making preferences. In addition, participants noted that having a navigator or peer counseling available would help with the decision-making process. One participant suggested that someone to help a patient “come up with [a] pros and cons list” “given your age and your situation” would have been helpful.

*Information preferences*: Multiple participants commented on the “information overload” they experienced, especially at the beginning when they were “in the middle of the hurricane.” One participant mentioned telling the nurse that “I just don’t understand it” and after that she “explained things a lot better.” They also mentioned the preference for including more details in patient education about the rationale for the combination treatment. Multiple participants noted having a good understanding of why OFS was recommended for them, but others reported “still [having] some confusion.”

Another theme was the need for more detailed education about administration of the injectable medication, which was mentioned by multiple patients taking OFS and who had discontinued it early. Some noted that for some patients, “not knowing what it actually looks like and will be like…is helpful for them,” but for others “knowing exactly what to expect…is helpful to them in terms of preparation.” Others commented on the critical need for “strategies for managing, coping with the pain” from the injections.

*Information needs of patients under 40*: Almost all of the 10 patients under age 40 remarked on the need for additional support because of relative lack of information about optimal treatment for very young women with breast cancer, the impact on life issues such as family planning, and the concern about “the long-term impacts of not having estrogen in your body.” They particularly noted the benefit of communicating with others also diagnosed at a young age since their “experiences are probably more similar.”

### Integrated analysis of qualitative and quantitative data

Overall, analysis of the integrated quantitative and qualitative data aligned with qualitative findings but revealed some potential areas for future hypothesis testing (Table 5). For example, those patients who reported a high degree of shared decision-making (SDM-9 scaled score > 75) aligned with the qualitative theme of explicit trust in their clinical team. However, almost half of the patients who scored highly for SDM and the qualitative theme of extensive discussion of treatment decision-making still reported a high level of concerns about ET based on the BMQ scale, which may suggest that high SDM and extensive discussion between patient and provider(s) are themselves not enough to allay the medication concerns of patients.

**Table 5:** Joint display demonstrating alignment between quantitative and qualitative results

Quantitative results	Qualitative results	Mixed methods inferences
Shared decision-making (SDM)-Q-9		
Higher SDM (SDM-Q-9 scaled score > 75)	Respondents reported a high degree of discussion, provider responsiveness to questions and feeling like they were a partner in the decision-making “I think anything that I’ve asked her, she would answer…And I think that she gave me the information and then allowed me to make my own decision instead of trying to push something on me” (8370)	Respondents with high SDM scores reported feeling like an informed partner, while respondents with lower SDM described feeling less like a partner and receiving little to no information on ET or alternatives
Lower SDM (SDM-Q-9 scaled score < 75)	Respondents had a greater tendency to report little discussion, that they received little information or feeling that their input was valued in the decision-making “It would just be nice to understand it more …from a one-on-one [with the] physician standpoint instead of reading stuff online and then trying piece it all together by myself…” (8343)	
Beliefs about medication questionnaire (BMQ)		
Moderate-to High weighing of BMQ Necessity over Concern (> 4 to 16)	Respondents described their side effects as “manageable.” “More irritations and annoyances…but they’re tolerable and manageable and I’ve learned to navigate around them or with them in life, so that they don’t impact the quality of life.” (0486)	Respondents with moderate to high necessity scores expressed more feelings of self-efficacy in managing their side effects than those who weighed necessity lower
Low-to-High (> 2 to 10) weighing of BMQ Concern over Necessity	Respondents voiced two types of concerns: a general wariness of taking medication and concern about the harmful long-term effects of ET. Many with these concerns had high shared decision-making scores “I mean, [I’m] very trusting…she’s [the oncologist] very aware of my priorities, my goals, and I think that informs her treatment plan of me…I just don’t like medication and putting it in my body… most women who are receiving [ET] are 20 to 30 years older than me…So a lot of the data…around long-term side effects is actually focused on those women…I am worried about that.” (2949)	Concerns reflected a distrust of medication and possible harm from medication, but this was not always accompanied by a lack of shared decision-making or trust in the provider

Participant numbers are given in parentheses

Those who reported higher levels of ET necessity compared to concerns on the BMQ described receiving the injections less negatively. In addition, those who expressed self-efficacy in managing their side effects from OFS, including stating they could mitigate them and actively engaging the support of their care team in doing so, tended to rate concerns about ET lower on the BMQ and tended to be still receiving the injections.

## Discussion

Results from the large, multinational SOFT and TEXT clinical trials clearly demonstrated improved breast cancer outcomes for young women at increased risk of recurrence of hormone receptor-positive breast cancer who take OFS [5, 6]. However, although rates of OFS use have increased somewhat since the initial trial results were published a decade ago, real-world studies suggest that only about 50% of eligible patients are taking OFS injections, with a low percentage of patients who undergo upfront BSO [11]. In this mixed methods study, we sought to identify patient-related reasons for incomplete initiation of and persistence with this effective therapy.

Qualitative themes were identified for each specific treatment cohort and across all cohorts. For those taking OFS injections, reducing the risk of cancer recurrence was the primary theme. Conversely, for many who stopped receiving the injections, permanence with surgical ablation was preferred, so treatment of OFS injections was often intended to be a temporary measure. However, across all cohorts, concern about the impact of short- and long-term side effects on quality of life was highly evident, especially in light of increasing public awareness of the protective functions estrogen performs in the body.

A prior qualitative study of oncologists highlighted both concerns about tolerability of OFS and patient hesitancy as reasons to forgo recommending treatment with OFS [16]. In contrast, a theme of our interview-based study of patients who were offered OFS by their oncologists demonstrated that most of those who took OFS injections experienced only mild toxicity. Many who discontinued OFS injections did so due to a stated preference to undergo BSO, as opposed to because of side effects. However, a substantial minority of both those who continue to take OFS and those who discontinued it early remain concerned about toxicity of therapy, especially long-term and late effects. Even those who reported high levels of shared decision-making and trust in their treatment team reported ongoing concern about the effects of adjuvant ET and OFS. These themes underscore that further research into ways to mitigate ET toxicity, especially for young women with breast cancer, remains critically important. Identifying effective methods for providing balanced education about benefits and toxicity from ET, including OFS, to patients may help support their decision-making as well as persistence with treatment.

In addition to reasons underlying decisions to initiate treatment with OFS, the interviewees highlighted education and support they wish they had received. A major theme was desire for detailed information to be provided about the injections, rather than either being surprised at the time of the initial injection or feeling that they had to seek out information on their own. In addition, there was interest in receiving more support and information from lay/nurse navigators or peer mentors both at the time of treatment decision-making and after the initial treatment period, once feelings of information overload had subsided. This highlights the critical need for development of patient-centered, reliable information that can support patients through the adjuvant ET and OFS decision-making process, ideally via a tailored approach that is aligned with individual patients’ preferences regarding medical shared decision-making and education. For example, use of a decision-support tool, the interactive iCanDecide website, tailored to patient preferences in the setting of breast cancer surgical decision-making was found to increase rates of high-quality decision-making compared to a static, non-tailored version [17].

For our study, identifying patients who had a discussion about OFS with their oncologist and subsequently declined treatment with OFS was challenging because of the inability to easily identify them from the electronic medical record. Of those who were interviewed, the population was heterogeneous. Some declined OFS therapy because of concerns about medication, while, others opted against initiating OFS because of a lack of strong recommendation by their oncologist. In addition, in some cases the patient started on treatment with tamoxifen with the plan to consider adding OFS in the future. The latter approach has been formally tested in a randomized clinical trial of premenopausal patients [18], and in a large survey it was reported as a commonly used approach by about 25% of oncologists [19].

The strengths of our analysis include heterogeneity of the participants, enabling elicitation of perspectives from those who have continued to take OFS injections, those who initiated the treatment and then stopped, and those who had a discussion about OFS but never started the injections. In addition, we elicited information through both qualitative interviews and quantitative questionnaires, which we analyzed separately and in an integrated, mixed methods dataset. This information can be used to develop approaches to support patients both during the decision-making process and following initiation of therapy. In contrast to those who took OFS injections, for whom we could systematically search the electronic medical record for patients who had goserelin or leuprolide therapy plans, our ability to identify potential participants who opted not to take OFS was limited to clinician identification or self-referral. Although we reached thematic saturation for the other two cohorts, the number of participants was small, limiting statistical power for identifying statistically significant associations and differences. Since almost all participants were from a single institution, the findings related to the need for additional educational resources may reflect the specific educational processes in place at that comprehensive cancer center and may not be generalizable across practice sites. We also did not capture which patients underwent BSO or were known to have pathogenic variants that predisposed them to increased risk of ovarian cancer, either of which may have influenced their perceptions of OFS. Finally, this study was limited to input from patients, not clinicians, although others have reported oncologists’ perspectives on the use of OFS therapy [16, 19]. Importantly, it is critical to understand the patient perspective when recommending treatments that can potentially cause both short- and long-term toxicity.

In summary, premenopausal patients with breast cancer varied in reasons for initiating and discontinuing OFS injections depending on perceived benefits and risks of treatment. Providing support to patients that is tailored to their decision-making preferences and needs, both at the time of the actual decision-making as well as during the treatment period, may increase both uptake of OFS therapy as well as persistence with adjuvant ET. Input from patients and clinicians will be critical for developing high-quality interventions to support decision-making for younger patients with breast cancer who are considering OFS as part of their treatment regimen.

## Data Availability

The datasets generated during and/or analysed during the current study are not publicly available but are available from the corresponding author on reasonable request.
